# Radiosensitising effect of iron oxide‐gold nanocomplex for electron beam therapy of melanoma in vivo by magnetic targeting

**DOI:** 10.1049/nbt2.12129

**Published:** 2023-04-21

**Authors:** Mahshad Mohamadkazem, Ali Neshastehriz, Seyed Mohammad Amini, Ali Moshiri, Atousa Janzadeh

**Affiliations:** ^1^ Radiation Biology Research Center Iran University of Medical Science (IUMS) Tehran Iran; ^2^ Radiation Science Department Iran University of Medical Science (IUMS) Tehran Iran

**Keywords:** EBRT, magnetic targeting, melanoma, nanoparticles, radiosensitiser

## Abstract

Melanoma is a dangerous type of skin cancer sometimes treated with radiotherapy. However, it induces damage to the surrounding healthy tissue and possibly further away areas. Therefore, it is necessary to give a lower dose to the patient with targeted therapy. In this study, the radio‐sensitising effect of gold‐coated iron oxide nanoparticles on electron beam radiotherapy of a melanoma tumour with magnetic targeting in a mouse model was investigated. Gold‐coated iron oxide nanoparticles were prepared in a steady procedure. The melanoma tumour model was induced in mice. Animals were divided into five groups: (1) normal; (2) melanoma; (3) gold‐coated iron oxide nanoparticles alone; (4) electron beam radiotherapy; (5) electron beam radiotherapy plus gold‐coated iron oxide nanoparticles. The magnet was placed on the tumour site for 2 h. The tumours were then exposed to 6 MeV electron beam radiotherapy for a dose of 8 Gy. Inductively coupled plasma optical emission spectrometry test, hematoxylin and eosin staining, and enzyme‐linked immunosorbent assay blood test were also performed. Gold‐coated iron oxide nanoparticles with magnetic targeting before electron beam radiotherapy reduced the growth of the tumour compared to the control group. Blood tests did not show any significant toxicity. Deposition of nanoparticles was more in the tumour and spleen tissue and to a lesser extent in the liver, kidney, and lung tissues. The synergistic effect of nanoparticles administered by the intraperitoneal route and then concentrated into the tumour area by application of an external permanent magnet, before delivery of the electron beam radiotherapy improved the overall cancer treatment outcome and prevented metal distribution side effects.

## Graphical abstract

The gold‐coated iron oxide nanoparticles were synthesised and characterised. A magnetic field was applied for the physical delivery of synthesised nanoparticles in the melanoma tumour in a mouse model. The radio sensitising effect of the nanoparticles on electron beam radiotherapy of the melanoma tumour was investigated.

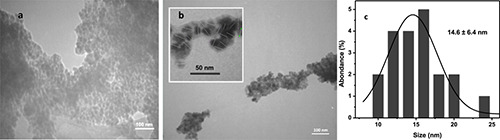

## 1 INTRODUCTION

Cutaneous melanoma destroys a large number of patients' life worldwide every year. The incidence and mortality of this rapidly progressive disease, vary greatly worldwide depending on access to early diagnosis and primary care [1]. Different treatment methods have been applied for this disease such as surgery to remove the tumour, radiation, immunotherapy, targeted therapy, or chemotherapy, each of which has its advantages and disadvantages, but overall, the treatment is difficult [2, 3].

In some studies, electron beam radiotherapy (EBRT) has shown good results. Probably because electrons have a short range and their maximum penetration depth is about 2–3 cm. Therefore, EBRT can apply a uniform dose to the tumour area without any extra radiation reaching the surrounding healthy tissue or deeper tissues [4, 5]. However, because tumour and healthy tissue are adjacents, normal tissue will be affected to some extent. For example, acute complications in skin tissue include arrhythmia, inflammation and scaling, as well as chronic complications such as fibrosis of the skin and underlying muscles [6].

The use of elements with a high atomic number can enhance the effect of ionising radiation. If these elements could be concentrated within the tumour, they would increase the absorption of ionising radiation compared to the surrounding tissues, thus enhancing the anti‐tumour effect [7]. Among materials containing elements with a high atomic number, metal nanoparticles have been widely investigated. Gold and iron oxide nanoparticles have both been used in new cancer treatments. Because these nanoparticles, in addition to being very small in size, have a high atomic number and a high photon absorption cross‐section, they can increase the effect of radiation and cause the emission of secondary electrons (Auger electron radiation), thereby inducing more apoptosis in tumour cells and more tumour destruction [8]. They can also act as contrast agents in x‐ray and magnetic resistance (MR) imaging approaches [9, 10]. Gold‐coated iron oxide nanoparticles (Au@IONPs) can increase radiation‐induced cell damage by triggering chemical reactions that produce cytotoxic reactive oxygen species (ROS) [11]. In vitro studies on the radiation‐sensitising effects of gold nanoparticles in the treatment of melanoma have been performed. According to these results, after irradiation with different doses of electrons delivered to the cells, the groups that received nanoparticles before irradiation showed a higher percentage of cell death [12, 13, 14].

In the present study, the Au@IONPs have been applied for the EBRT treatment of melanoma cancer in an animal model. The C57BL/6 mouse B16F10 melanoma model with some similarity to human melanoma tumours [15], was applied for the study. The iron content could be used as a contrast agent in MR imaging. In addition, these particles could be manipulated using an external magnet placed over the tumour area to increase the accumulation of nanoparticles in the tumour tissue. We were attempting to take full advantage of Au@IONPs to investigate the effect of its radiation sensitisation on melanoma tumours irradiated with electron beams with magnetic targeting. Next, the distribution of the Au@IONPs in various tissues of the body, such as the liver, brain, and spleen was examined by inductively coupled plasma optical emission spectrometry (ICP‐OES), and blood biochemistry was performed using enzyme‐linked immunosorbent assay (ELISA) kits. For histological evaluation, samples of different animals' organs were stained by hematoxylin and eosin (H&E).

## 2 MATERIALS AND METHODS

### 2.1 Synthesis and characterisations of Au@IONPs

Au@IONPs were synthesised in a step‐by‐step procedure similar to our previous report [16]. First, iron oxide NPs were synthesised by the co‐precipitation technique. Shortly, 12.5 ml of NH_4_OH (2 M) was added dropwise to a 2:1 solution of FeCl_3_.6H_2_O and FeCl_2_.4H_2_O under extreme stirring leading to sedimentation of iron oxide NPs. The formed sediment was separated by an external magnetic field, then washed and dispersed in a solution of toluene (40%)/dimethylformamide (60%). By adding 5 ml of (3‐aminopropyl)triethoxysilane and refluxing for 24 h, the iron oxide NPs were functionalised. Again, the NPs were washed with methanol, utilising a magnetic field. To create a gold coating, first, a seed solution of 3 nm gold NPs was synthesised according to the previously presented methods [17]. For the immobilisation of the synthesised gold seeds onto the amine‐functionalised magnetic NPs, 4 ml of amine‐functionalised magnetic NPs (51.5 mM Fe) was added dropwise to a 25 ml solution of gold seeds (0.198 mM Au). After 2 h, the Au NPs were stabilised onto the surface of the iron oxide NPs. Finally, the gold coating was generated by adding H(AuCl_4_)·3H_2_O (15 ml, 2.15 mM) and subsequent ascorbic acid (0.4 ml, 78.9 mM) under sonication. The unreacted particles and ions were washed, and the remained Au@IONPs were dispersed in 15 ml of deionized water.

The size and morphology of the Au@IONPs were investigated by transmission electron microscopy (TEM, Zeiss EM 900, Germany). For calculation of the size distribution of the Au@IONPs, obtained micrographs were analysed by Digital Micrograph software. The hydrodynamic diameter of the Au@IONPs was studied by a nanoparticle measurement system (Nano‐flex, Analytik, UK). The surface charge/Zeta potential of the particles was also verified by Malvern Nanosizer (Malvern Instrument, UK) in natural pHs. The pattern of X‐ray powder diffraction (XRD X’Pert Pro MPD‐PANalytical [Cambridge, UK], Cu K*λ*, 40 kV, 30 mA) was acquired after 300 μl of Au@IONPs sample was cast on a silicon substrate. The UV–Vis spectroscopy (Rayleigh UV‐1601 instrument, Beijing, China) was also applied for analysis of the plasmonic peak of the Au@IONPs. ICP‐AES (VISTA‐PRO, Varian, Australia) has been applied for the determination of the Au/Fe ion concentration.

### 2.2 In vitro study

#### 2.2.1 Culture of melanoma cells

The B16F10 mouse melanoma cell line was obtained from the Pasteur Institute of Iran. Cells were cultured in Dulbecco's modified eagle medium medium containing 10% fetal bovine serum and 1% penicillin at 37°C and 5% carbon dioxide. After three cell passages, they were separated from the flask by trypsin solution. Two million cells were suspended in 200 μl of bovine foetal serum and prepared for subcutaneous injection into the right thigh of each mouse.

#### 2.2.2 In vivo experiments

Thirty male C57BL/6 mice aged about 6–8 weeks and weighing about 20 g were obtained from the Pasteur Institute of Iran. The animals were kept in well‐ventilated cages at the Center for Experimental and Comparative Studies of Iran University of Medical Sciences. Six mice were kept in each cage and had access to adequate water and food during the study. The ambient temperature was about 21 ± 1°C and relative humidity was set at 45%–60%. All animal experiments were performed following guidelines set by the Committee for Basic Animal Care. The ethics protocol number is IR.IUMS.REC.1397.1094 was approved by the Research Council of Iran University of Medical Science.

### 2.3 Study design

Experiments were commenced after the tumour volume reached 100 mm^3^. The animals were randomly divided into five groups of six mice: (1) normal mice, without melanoma tumour and with no treatment; (2) control group, with untreated tumour; (3) Au@IONPs alone. Intraperitoneal injection of 0.1 ml nanoparticle suspension with the concentration, Au 50 μg/ml and Fe 100 μg/ml [18]. (4) EBRT alone; tumours were exposed to a single dose of 8 Gy with 6 MeV energy; (5) combination Au@IONPs plus magnet plus EBRT. After the intraperitoneal injection of nanoparticles, a 0.4 T magnet was placed on the tumour site for 2 h [18]. Then the mice were exposed to EBRT with 6 MeV energy and 8 Gy dose.

Experiments were commenced after the tumour volume reached 100 mm^3^. The tumour volume of the mice was measured every 2 days for 3 weeks after starting treatment. Tumour volume was calculated based on (width)^2^ × length. Mouse body weight and general condition were also monitored. The experiments ended before the maximum tumour volume of the main treatment group reach 2000 mm^3^, at the end of the third week, the animals were euthanised by injection of 100 mg/kg ketamine, then 2 ml of blood samples from the left ventricle of each mouse were collected and the liver tissue, kidney, lung, spleen, brain, and tumour were removed for further studies.

### 2.4 Animal radiation setup

After anaesthesia, the animals were fixed on an inflexible plate so that the animals' right foot (tumour site) was in the radiation field. Then they were placed at a distance of 1 m from the radiation source and electron irradiation was performed with the mentioned conditions.

### 2.5 Histological studies

Liver, kidney, lung, and tumour tissues were selected for H&E staining in three randomly selected mice from each group. The method was described in previous studies [19]. Briefly, the tissues were placed in 10% paraformaldehyde for fixation and then immersed in alcohol, and then in xylol for dehydration. Then they were embedded in paraffin. Using a microtome device, sections with a thickness of 5 μm were prepared from a paraffin block and stained with H&E. Three sections were selected from each tissue and photographed with a 10× and 40× magnification light microscope [20].

### 2.6 Blood chemistry study

To evaluate the effect of treatments on organ function, the levels of P, Na, K, serum glutamic‐oxaloacetic transaminase (SGOT), serum glutamic‐pyruvic transaminase (SGPT), blood urea nitrogen (BUN), C‐reactive protein (CRP), and creatinine in animal blood samples were evaluated. For this purpose, blood serum was isolated using centrifugation and evaluated by ELISA kits [21]. The kits used for these tests were provided by Delta Darman Company, Iran. The characteristics of the kits were included in Table 1.

**TABLE 1:** Characteristics of kits used in the study.

Factor	Creatinine	Urea	SGOT	SGPT	Na	K	Ph	CRP
Kit number	98011	1930120	1590520	1540420	1660319	1660319	1880420	1701020
Kit brand	Pars.Azmun	Delta‐DP	Delta‐DP	Delta‐DP	Delta‐DP	Delta‐DP	Delta‐DP	Delta‐DP

### 2.7 Tumour accumulation and tissue distribution of Au@IONPs

The distribution of nanoparticles in the liver, kidney, spleen, lung, brain, and tumour tissues was evaluated in the two groups receiving nanoparticles (Au@IONPs, Au@IONPs plus EBRT) by the ICP‐OES. The tissues were weighed after removal and dissolved in an aqua regia (HCl:HNO_3_, 3:1). Then ICP‐OES test was performed to measure the amount of iron and gold. The procedure was similar to previous studies [22, 23].

### 2.8 Statistical methods

Statistical analysis was performed using Prism 8.1 software. One‐way analysis of variance tests were used to evaluate the significance of the data. *p* value < 0.05 was considered significant.

## 3 RESULTS

### 3.1 Synthesis of Au@IONPs

The Au@IONPs were synthesised and characterised in the exact procedure of our previous studies [16]. The full characterisations of the Au@IONPs were performed in our previous studies which include an investigation of the size, hydrodynamic diameter, and zeta potential of the particles. Also, the characteristic plasmonic absorption spectra of gold shell and the crystalline structure of NPs were observed previously [16, 24]. However, to ensure the proper characteristics of the Au@IONPs used in this study, the nanoparticles were characterised with UV–Vis spectroscopy, XRD, TEM, and dynamic light scattering (DLS) before in vivo investigations.

**FIGURE 1:**
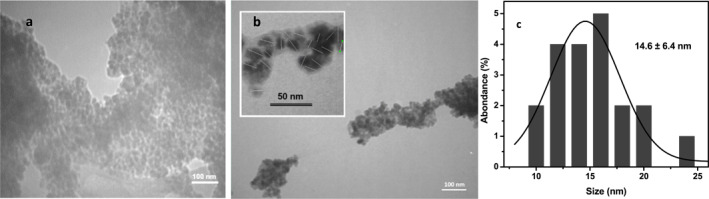
TEM micrographs of the synthesised iron oxide nanoparticle (a), gold‐coated iron oxide nanoparticle (Au@IONPs) (b) and the obtained size distribution histogram (c). The Au@IONPs have been marked with a separate white dash micrograph in the up‐left of figure (b).

**FIGURE 2:**
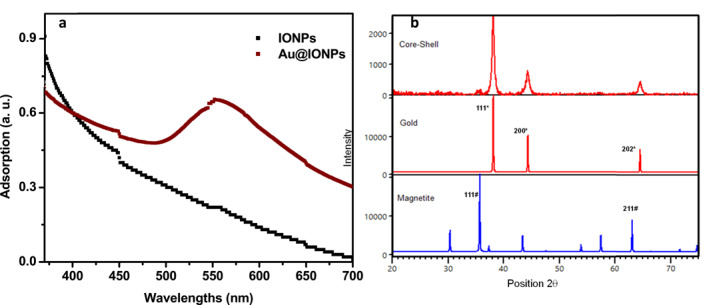
UV–Vis spectra of IONP and Au@IONPs (a), the plasmonic peak of the gold shell was observed at 565 nm. XRD pattern of Au@IONPs. XRD pattern for Au@IONPs based on JCPDS database (b).

The semi‐spherical shape and core–shell structure of nanoparticles were observed in TEM micrographs (Figure 1). As a result of the magnetic field of the electron microscope that affects the ferromagnetic Au@IONPs, aggregates are observed. This aggregation was observed for both iron oxide and gold coated iron oxide nanoparticles (Figure 1a,b). However, the separate Au@IONPs are expected to have an average diameter of 14.6 ± 6.4 nm. The UV–Vis spectra of the synthesised nanoparticles represents a plasmonic peak at 565 nm approximately (Figure 2a). Also, the XRD pattern was analysed based on Joint Committee on Powder Diffraction Standards (JCPDS) database, and the strongest peak was marked based on JCPDS card numbers 96‐900‐8464 (Au cubic), and 96‐101‐1033 (FeO cubic).

**FIGURE 3:**
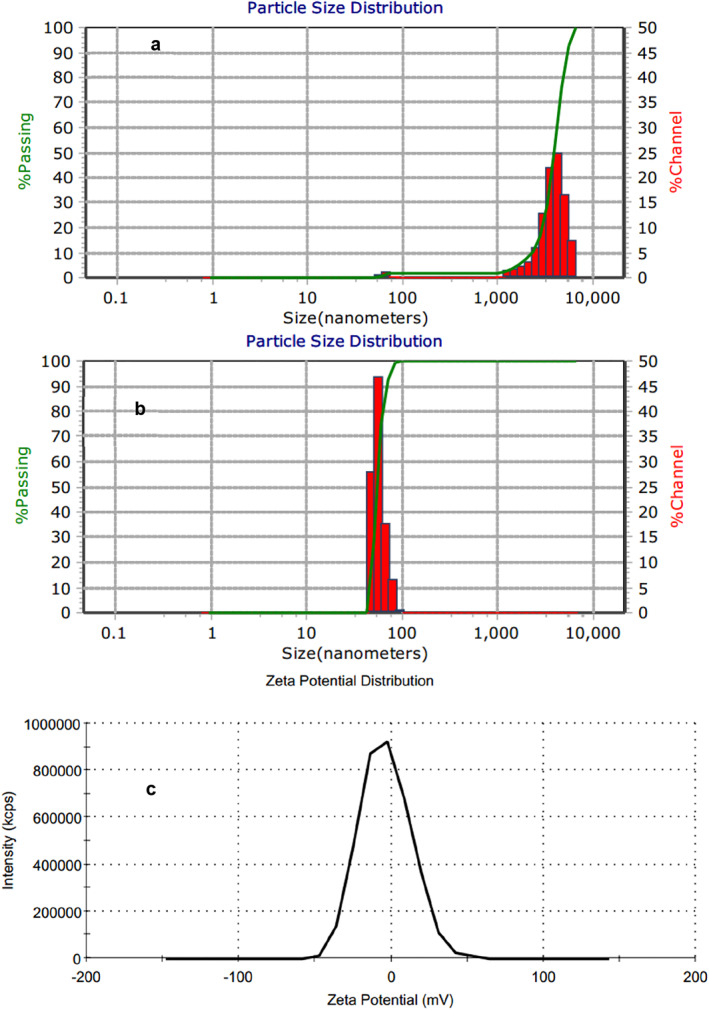
Hydrodynamic size distribution based on intensity (a) and number (b) of the synthesised gold‐coated iron oxide (Au@IONPs) nanocomplex. The average hydrodynamic diameter of the Au@IONPs is 55.2 nm. The average zeta potential of the Au@IONPs is −3.8 mV (c).

According to the results of the DLS test, the hydrodynamic diameter of the Au@IONPs was 55.2 nm (Figure 3a,b). Based on the results of the zeta potential investigation, the surface charge potential of nanoparticles was measured at −3.8 mV (Figure 3c).

### 3.2 Tumour growth measurement

**FIGURE 4:**
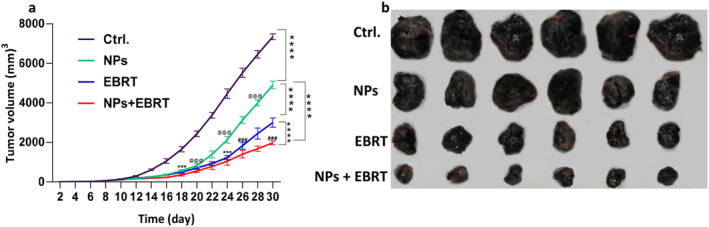
In vivo assessment of antitumour effects of gold‐coated iron oxide Au@IONPs nanoparticles. (a) Tumour volume change 21 days after treatment ****p < 0.0001 versus control group, @@@*p* < 0.0001 between NPs and NPs + EBRT, ###*p* < 0.0001 between EBRT and NPs + EBRT. (b) Representative photographs of tumour samples after various treatments. Ctrl., control; EBRT, electron beam radiation therapy; NPs, nanoparticles.

In the third week after treatment, inhibition of tumour growth was observed in all the treatment groups compared to the control group. On day 30 of the study, the mice of tumour growth inhibition compared to the control group was 33% in the Au@IONPs group (tumour volume of 4900 mm^3^ compared to 6500 mm^3^) and 57% in the EBRT group (3000 mm^3^ tumour volume). The tumour volume was 74% lower in the combination Au@IONPs + EBRT group (1900 mm^3^ tumour volume). Tumour growth was significantly inhibited in the treatment groups compared to the control group (*p* < 0.0001). From day 18 after treatment, a significant difference was observed between the treatment and control groups. On day 20, the difference between the Au@IONPs alone and the combination group was significant (*p* < 0.0001). Also, from day 26 to 30, a significant difference was observed between the EBRT group and the combination group (*p* < 0.0001) (Figure 4).

### 3.3 Animal body weight changes

**FIGURE 5:**
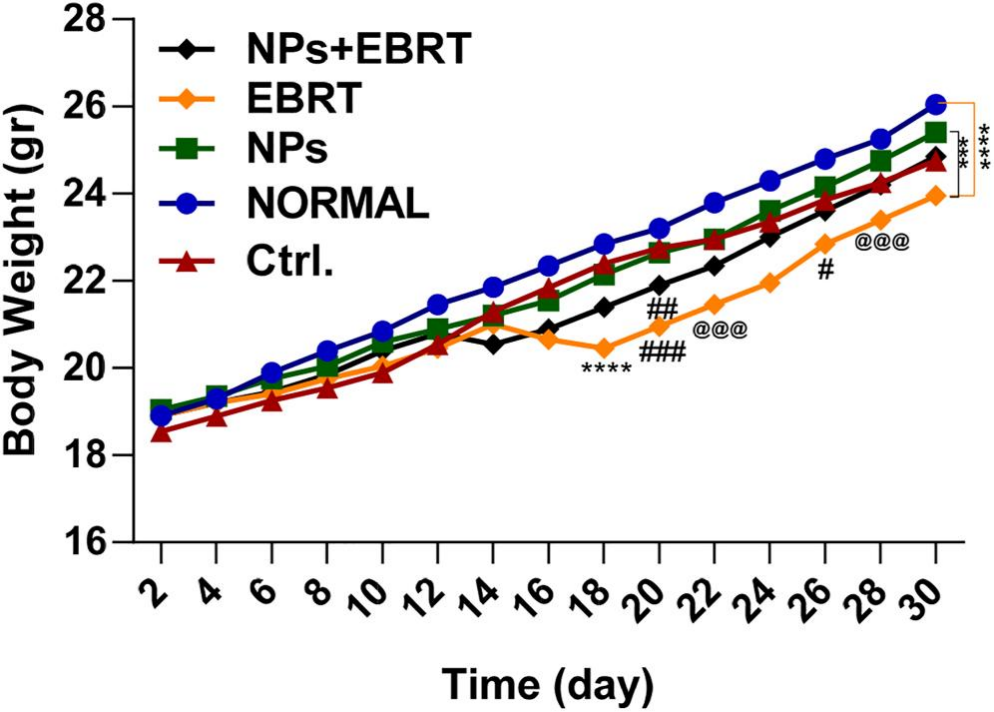
The body weight change of C57BL/6 tumour‐bearing mice as a function of time post‐treatment. ***p < 0.001, ****p < 0.0001 versus normal, ###*p* < 0.001 ##*p* < 0.01 #*p* < 0.05 versus control, @@@ *p* < 0.001 between NP and EBRT. Ctrl., control; EBRT, electron beam radiation therapy; NP, nanoparticle; Np + EBRT, nanoparticle + electron beam radiation therapy.

In all the groups, the body weight of the mice increased over time. However, in two groups EBRT and EBRT plus nanoparticles, a slight weight loss was observed in the first week after treatment. However, the weight of mice in these groups increased again over time and returned to normal levels. The mean weight of animals in the third week after treatment in the nanoparticle group was not different from the normal group. However, in the control, EBRT, and combination groups, the mean weight of the mice was lower than normal. The lowest weight was the EBRT group with 23.9 g. The mean weight of the control, combination, nanoparticle, and normal groups were 24.7, 24.9, 25.4, and 26 g respectively (Figure 5).

### 3.4 Biodistribution of Au@IONPs

**FIGURE 6:**
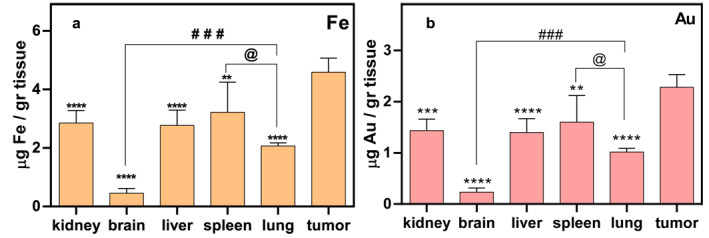
Inductively coupled plasma optical emission spectrometry (ICP‐OES) analysis of essential organs to demonstrate accumulation of Au@IONPs 3 weeks after the intraperitoneal injection. (a) The Fe content of various organs (b) the Au content of various organs. ****p < 0.0001, ***p < 0.001, **p < 0.01, *p < 0.05 in comparison to the tumour, @*p* < 0.05 between the lung and brain, ###*p* < 0.001 between the lung and brain.

Changes in nanoparticle deposition were observed in all tissues, especially the liver, kidney, spleen, and tumour tissues. These amounts were lower in the brain than in other tissues, and the difference in the brain compared to the liver, kidney, spleen, and lung tissues were significant (*p* < 0.0001). In addition to the tumour, deposition of nanoparticles was observed in the spleen tissue and to a lesser extent in the liver, kidney, and lung tissues. These changes were significant in tumour tissue compared to other tissues (*p* < 0.0001), but not significant in kidney, liver, or spleen tissues (Figure 6).

### 3.5 Toxicology analysis

**FIGURE 7:**
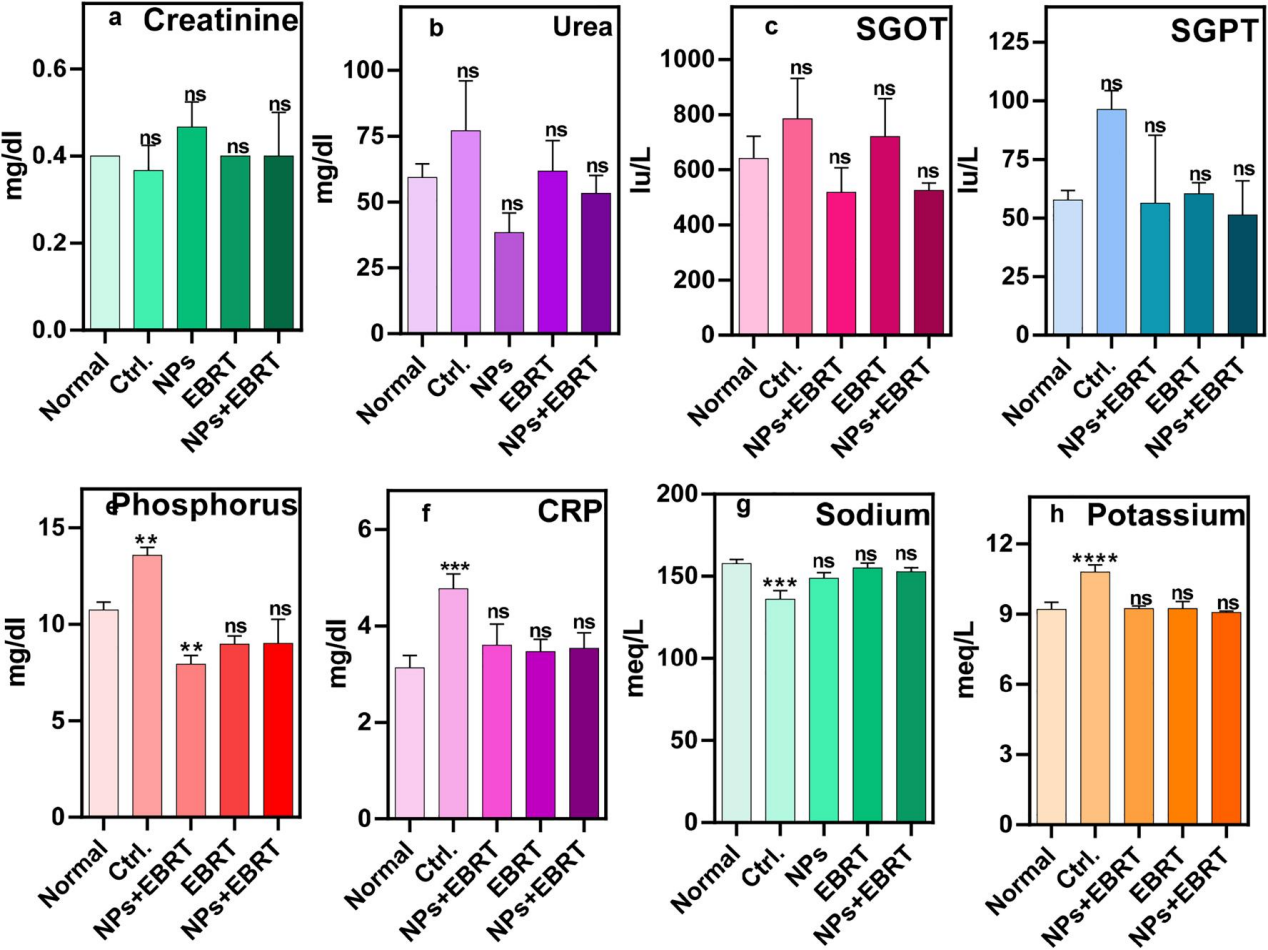
Blood test results as an indicator of organ's function: (a) urea (b) creatinine (c) SGOT (d) SGPT (e) CRP (f) phosphorus (g) sodium (h) potassium. CONT, control; CRP, C‐reactive protein; EBRT, electron beam radiation therapy; NP, nanoparticle; Np + EBRT, nanoparticle + electron beam radiation therapy; SGOT, serum glutamic oxaloacetic transaminase; SGPT, serum glutamic pyruvic transaminase. ****p < 0.0001, ***p < 0.001, **p < 0.01, *p < 0.05 in comparison to normal group.

Using ELISA tests, the levels of BUN, creatinine, SGOT, SGPT, P, Na, K, and CRP in serum samples were measured. Based on the results, creatinine and urea levels were almost the same in all the groups and no significant difference was observed in the renal function between the treatment groups (Figure 7a,b) (*p* value > 0.05).

Any changes in SGOT and SGPT levels, which reflect liver function between the control groups compared and the normal group were not significant (*p* value > 0.05) (Figure 7c,d). There was also no significant difference in liver function between treatment groups (*p* value > 0.05). The mean level of phosphorus in the blood samples of mice after 3 weeks from the start of treatment was 10.7 mg/dl in the normal group, 13.6 mg/dl in the tumour group, 7.9 mg/dl in the nanoparticles group, 9 mg/dl in EBRT group and combination group. These differences were significant between the normal group, and the control group or nanoparticle group (*p* < 0.001) (Figure 7e). The results of the inflammatory marker CRP did not show a significant difference between the treatment groups. However, significant differences were observed between the normal group and the control group. The CRP level in the normal group was 3.1 mg/dl and in the tumour group was 4.8 mg/dl (Figure 7f, *p* < 0.001).

The amount of sodium in the blood sample of the treatment groups at 3 weeks after treatment was not significantly different from the normal group *p* > 0.05. The mean sodium level was 158 meq/dl in the normal group and 136 meq/dl in the tumour group. This value was lower in the control group than in the normal group, which was significant (Figure 7g, *p* < 0.0001).

The mean level of potassium in the blood of mice in the third week of the study was 9.2 meq/dl in the normal group and 10.8 meq/dl in the untreated tumour group, which was statistically significant compared to the normal group (*p* < 0.0001). No significant differences were observed between the other treatment groups and the normal group (Figure 7h, *p* > 0.05).

### 3.6 Histological staining

The following reports were provided by a pathologist who examined the H&E stained sections.

#### 3.6.1 Kidney

**FIGURE 8:**
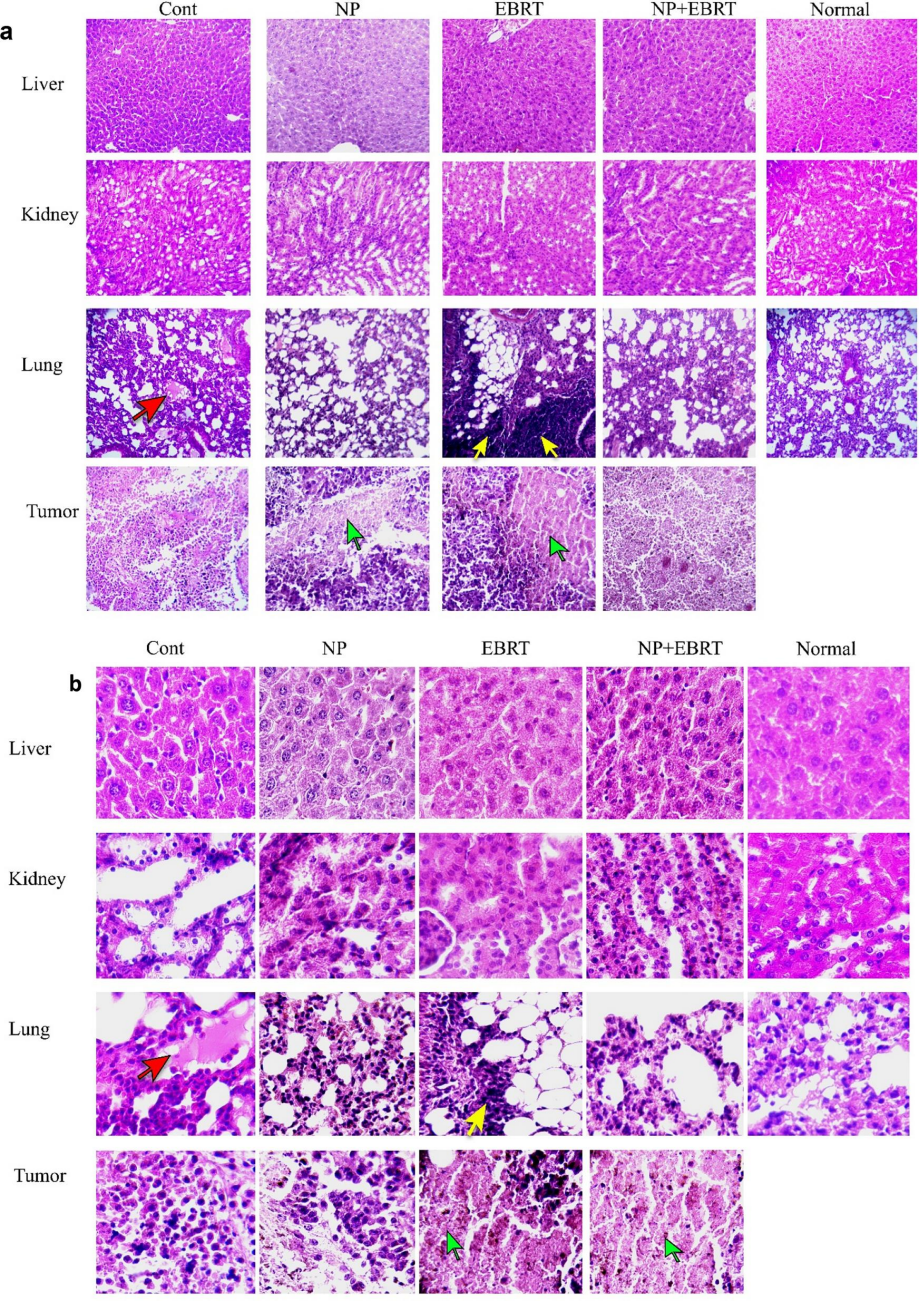
Histological evaluation. Representative hematoxylin and eosin images of liver, kidney, lung, and tumour, (a) 10× magnification (b) 40× magnification (Red arrow, fibrosis; yellow arrow, inflammations; green arrow, necrotic tissue).

In the control group, apoptosis, fibrosis, atrophy of kidney cells, and glomerular dilatation were observed. In this group, the kidney capsule was slightly atrophied, however, no significant infection or inflammation was seen. No inflammation was seen in the nanoparticle group, but scattered necrotic cells were seen. In the EBRT group, the kidney tissue was dense. There was some necrosis around the renal glomeruli, but no inflammation was seen. In the combined group, hypercellular and hyperaemic kidneys were observed along with some necrosis, fibrosis, and polymorphonuclear neutrophil (PMN) cells (Figure 8a,b).

#### 3.6.2 Liver

No inflammation was seen in the control group. Dilatation of bile ducts and a limited number of necrotic cells were observed in the EBRT group, but no inflammatory cells were seen. However, this dilatation of bile ducts was not observed in the control group. In the nanoparticle group, as in the EBRT group, necrotic cells were seen among the hepatocytes. In the EBRT plus nanoparticles group, liver tissue did not show any inflammation, but PMN cells were observed around the blood vessels in the liver (Figure 8a,b).

#### 3.6.3 Lungs

In the control group, some hyperemia and hypertension were seen. Slight inflammation was observed in the lung tissue and the alveolar wall was thickened. Fibrin was observed in the alveoli and signs of metastasis were observed. The walls of the pulmonary arteries were also thickened. In the electron therapy group, inflammation was observed in lung tissue and PMN cells, and the walls of the alveoli were indicative of emphysema. In the nanoparticle group, a limited number of PMNs and fibrin cells were observed, which was not significant compared to the EBRT group. In the combination group, slight inflammation was seen, but was not significant. Also, necrosis and atelectasis of cells were observed (Figure 8a,b).

#### 3.6.4 Tumour tissue

The control group was hyperaemic and inhomogeneous. PMNs, necrotic and apoptotic cells were seen in the tumour tissue, and the tumour also had actively growing regions. In the EBRT group, the tumour tissue was very involved and completely inflamed. Plasma cells, macrophages, neutrophils, and lymphocytes were seen in the tumour tissue. The tissue had extensive necrosis. Necrosis and PMN cells were also observed in the nanoparticle group. The tissue also had fibrosis and hyperemia. In the combination group, severe inflammation and necrosis were observed in the tumour tissue. The cell density was very low and a large volume of tissue was necrotic, and the tumour tissue was almost destroyed and inactive. A large number of necrotic centres and PMN cells were observed (Figure 8a,b).

## 4 DISCUSSION

Melanoma is a serious type of skin cancer that is considered life‐threatening in a short time if untreated. Gold nanoparticles were applied as radiosensitisers for EBRT treatment of melanoma tumours by Chang et al. [25]. Twenty‐four hours before EBRT, nanoparticles were injected intravenously and then 25 Gy electrons were delivered into the melanoma tumours [4]. However, since 25 Gy is a high dose for animals, in the present study, a lower dose was used with a new type of nanoparticles with a core–shell structure containing iron in addition to gold.

The synthesis of Au@IONPs was characterised by different techniques. TEM micrographs represent semi‐spherical nanoparticles with a double contrast, which is caused by the difference in the two elements of iron and gold electron densities in the nanostructure. Also, the aggregation of the nanoparticles. The ferromagnetic structure of the Au@IONPs forces the nanoparticle to be aggregated on the grid surface during the TEM experiment. This aggregation was observed for both gold‐coated and uncoated iron oxide nanoparticles. So, we calculate the size of the nanoparticles that had clear borders with other nanoparticles. The size of such nanoparticles is demonstrated with a line drawing a dash through the Au@IONPs diameter (separated micrograph (top left) in Figure 1b). Schwaminger et al. also calculated the size distribution similarly. They declared the size of 10 nm for the gold‐coated magnetic NPs [26]. However, the provided TEM represent aggregated nanoparticles as a result of the magnetic force of the TEM experiment. Similar TEM micrographs were also reported for Au@IONPs by other research groups [26, 27, 28].

Their surface charge was measured at −3.8 mV, which indicates the good Au coating around IONPs. However, the amine‐functionalised IONPs represent a high positive value of zeta potential. The low value of zeta potential is causing the aggregation of the nanoparticles because of the lack of electrostatic repulsion [29]. According to the DLS results, the nanoparticle size was in the range of 40–60 nm, with a peak of 55 nm, which was higher than the TEM micrographs results. The diagram of intensity results (Figure 3a) represents two distinct peaks (65 nm, 3.8 μm) established on the scattering intensity of helium–neon laser rays. The dependence of Riley scattering approximation is highly dependent on particle diameter (R_s_d^6^) [30]. Then few aggregated or flocculated nanoparticles could lead to different intensity patterns. Thus a number histogram was represented in Figure 3b (hydrodynamic diameter: 55.2 nm).

Gold nanostructures with various shapes represents a surface plasmon resonance characteristic peak [31]. As represented in Figure 2a, the plasmonic peak of the gold shell is observed around 570 nm. IONPs do not have this characteristic plasmon peak, which indicates that IONPs are covered with a gold shell in Au@IONPs. The diffraction peaks of the Au@IONPs is marked in the selected JCPDS of gold and iron oxide. The XRD pattern demonstrated that the sample contain cubic strictures of both Au and iron oxide crystalline.

The therapeutic results showed that in all treatment groups, the growth of the mouse melanoma was reduced. In the Au@IONPs‐receiving group decrease in tumour growth was seen by more power. In the groups that received nanoparticles, this may be due to the increased permeability and decreased lymphatic drainage in tumour tissue known as enhanced permeation and retention (EPR) effect. This means that the entry and accumulation of nanoparticles within tumour tissue are higher than in healthy tissue. The accumulation within the tumour tissue could cause a gradual inhibition of growth, possibly by the production of free radicals, and disruption of cell repair. On the other hand, nanoparticles cannot penetrate normal tissue due to the tight endothelial cell junctions of normal capillaries (5–10 nm) [32]. This property could explain why the nanoparticles were effective in tumour tissue, however, did not have any destructive effect on healthy tissue.

Another specialty of the present therapy is that Au@IONPs entrapment in the tumor area by implementing an external magnetic field might prevent many side effects of Au@IONPs distribution in other organs [33, 34, 35]. The use of non‐targeted nanoparticles causes them to spread more systematically to other tissues, and this can increase side effects in healthy non‐target tissues. Including reproductive tissues that are very sensitive [35, 36]. While the use of the magnetic targeting technique in nanoparticle delivery causes more accumulation of nanoparticles in the tumour tissue and prevents their systematic spread. As a result of entrapment Au@IONPs, in the cellular environment undergo biodegradation, resulting in cellular reactions following the degradation of nanoparticles. For example, degraded nanoparticles may accumulate inside cells, leading to intracellular changes such as organ dysfunction. Or gene changes that cause severe toxicity [37]. The additional accumulation of iron nanoparticles produced by the application of the external magnetic field may have contributed to the targeted accumulation of nanoparticles in the tumour and increased the therapeutic effect of the Au@IONPs.

In general solid tumours are characterised by an increased EPR effect, however, this may not be considered for every tumour. It has been demonstrated that inorganic nanoparticles could interact with vascular endothelial cadherin and disrupt endothelial cell–cell interactions and induce leakiness in the vasculature [38]. This effect was called nanoparticles‐induced endothelial leakiness (NanoEL). The NanoEL effect of titanium dioxide, silica and gold nanoparticles caused a significant increase in intravasation and extravasation of tumour vasculature [39]. The NanoEL effect may be more effective in gold‐coated magnetic nanoparticles. Because it has already been reported that the application of an external magnetic field to accumulate the magnetic nanoparticles in tumour volume may disrupt the intercellular adherens junction protein vascular endothelial‐cadherin [40]. In the combination therapy, these properties were combined and caused more inhibition of tumour growth. A gold ion with a high atomic number can generate secondary electron radiation (Auger) under the influence of the electron beam to improve the effect of EBRT [8].

In the EBRT group, a decrease in tumour size was caused by necrosis and inhibition of cell division in the tumour tissue. In the EBRT group, DNA damage is caused by radiation, and the cells are arrested in the G2/M phase of the cell cycle. Also, the production of free radicals causes additional cell damage within the tumour [11, 41, 42]. Because of the radiation effect, the reduction of tumour size was more pronounced in the EBRT group than in the nanoparticle‐alone group.

From the 14th day after therapy onwards, a decrease in tumour size was observed in all the treatment groups compared to the control group, and from the 20th day, this difference intensified between the nanoparticle group and the combination therapy group. In combination therapy, the radiation‐sensitising effect of the Au@IONPs caused more cell damage in the tumour tissue. The combination therapy, which showed the greatest reduction in tumour size, demonstrated considerable necrosis within the tumour tissue, leading to more tumour growth inhibition. This finding suggests a synergistic effect between the nanoparticles and EBRT. Based on these results, we suggest that a combination of nanoparticles and EBRT is appropriate for inhibiting the growth of melanoma tumours [13, 14, 43, 44].

In this study, the body weight of the mice was also assessed during the treatment period [18, 22]. Mice in the control and nanoparticle groups showed a gradual weight gain. Nevertheless, in general, their weight was still significantly lower than the normal animals. Weight loss and cachexia are features of cancer, including melanoma. Cachexia is a cancer‐associated catabolic condition characterised by loss of muscle mass and a drop in body weight. Cachexia often occurs in patients with end‐stage neoplastic disease and is often an indirect cause of cancer death [45]. For this reason, the animals in the control group showed weight loss.

Weight loss in the EBRT group intensified from the 16th day after treatment, although the animals lost less weight than the control group. Radiotherapy has previously been shown to be associated with side effects such as loss of appetite, fatigue and, diarrhoea that can lead to weight loss [17, 46]. In clinical studies, it has been observed that weight loss due to radiotherapy can continue for up to 1 month after treatment, which could explain the continuation of the weight loss in our study [17, 47]. Treatment with NPs alone did not affect weight loss, but in NP + EBRT weight loss was also observed, which was probably due to the effect of radiotherapy.

Excessive deposition of gold and iron could cause serious problems for various organs. For this reason, the number of nanoparticles deposited in major organs, such as the liver, kidney, lungs, brain, spleen, and tumour was evaluated 3 weeks after treatment with the ICP assay. Based on the results, the amount of Au@IONPs deposited in the tumour tissue was higher than in other organs, which is encouraging for successful treatment. Additional accumulation of Au@IONPs in the tumour tissue could have been due to the magnetic targeting, in addition to other reasons, such as the EPR or NanoEL effect.

Accumulation of Au@IONPs was also observed in the liver, kidney, spleen, and lung tissues, in approximately similar amounts. The liver, spleen, and lungs are all known to be part of the reticuloendothelial system (RES). This suggests that nanoparticles could eventually be excreted from the body via the RES removal process. Also, the amount of Au@IONPs in brain tissue was very low, which suggests that the blood–brain barrier prevented the passage of nanoparticles into the brain, and moreover that the nanoparticles did not damage the blood–brain barrier itself. The number of nanoparticles deposited in liver, kidney, and spleen tissues were not significantly different. In a previous study, after analysing tissue samples, they reported that the number of nanoparticles in tumour tissue, liver, and spleen of animals was high [25, 48].

Controlling and reducing metastasis while at the same time inhibiting tumour growth, without any tissue damage to healthy tissue is the main goals of cancer treatment. To investigate any damage to normal tissue, H&E staining was performed on tissue samples prepared from different organs. After evaluating the stained tissue samples, no significant inflammation or involvement was observed in the liver tissue from all the different treatment groups. Although necrotic cells were observed in some samples, no significant increase in markers of kidney damage, such as urea and creatinine levels. These results indicate that the treatments did not have a significant effect on kidney function.

SGOT and SGPT, which are important markers of liver function, were also evaluated. In the control group, the level of SGPT was significantly increased compared to the normal group, which could be due to tumour metastasis to the liver. However, the levels of these markers in other treatment groups were not significantly different from the normal group. These results indicate that the treatments did not have any significant effect on liver function and could also prevent liver metastasis.

In the EBRT group, inflammation was observed in lung tissue, and especially in tumour tissue. Also, the lungs of the animals in the combination group showed a slight inflammation, which was much lower than in the EBRT alone group. Pneumonia is a known side effect of clinical electron beam therapy. But the important finding was that there was no significant difference between the CRP values indicating that the inflammation was no more than the normal group. The CRP levels increased only in the control group, indicating inflammation, probably due to cancer progression and possible metastasis [49]. Tumour growth can cause widespread tissue inflammation and therefore increase CRP levels [50, 51, 52]. CRP can also be an indicator of an immune response against tumour antigens [53]. In addition, there is evidence that cancer cells can increase the production of inflammatory proteins from infiltrating macrophages, which may explain the high concentration of CRP in cancer patients. Some cancer cells themselves have been shown to express CRP [54].

Also, the volume and growth of the tumour tissue in the combination group were largely well‐controlled, with a large part of the tumour containing necrotic cells, and most of the tumour tissue was inactivated. Analysis of the amount of phosphorus in the blood samples of mice showed that in all treatment groups, there was a decrease in the amount of phosphorus compared to the normal group. In the control group, P was increased compared to normal mice. The reduction of phosphorus in the nanoparticle group was also significant. Decreased phosphorus in the nanoparticle group could be due to more excretion by the kidneys. The increased phosphorus in the control group could be due to more growth of the tumour. In cancer and metastasis, the amount of phosphorus usually increases because tumour cells have a rapid turnover. They, therefore, require a constant source of phosphate, a nutrient essential for the synthesis of nucleic acids, phospholipids and high‐energy metabolites such as adenosine triphosphate. For this reason, high levels of phosphorus in the blood were considered to be a sign of progressive cancer growth in the mouse model [55, 56].

With regard to the sodium and potassium levels, we did not observe any significant difference between the treatment groups and the normal group. In the control group, without treatment, potassium levels were increased and sodium levels were decreased, which could indicate minor renal dysfunction. In general, electrolyte changes are observed in cases of cancer and metastasis, which can include changes in potassium and sodium levels. In this study, the decrease in sodium in the control group could be due to kidney metastasis or renal failure. Also, the increase in potassium in the control group could be due to cancer or damage to the renal parenchyma following metastasis or inappropriate renal excretion [57].

## 5 CONCLUSION

In summary, the gold–iron core–shell nanoparticles developed in this study displayed a radio‐sensitising property to potentiate the effects of electron beam therapy to inhibit the growth of melanoma tumours. The synergistic effect of nanoparticles administered by the intraperitoneal route and then concentrated into the tumour area by application of an external permanent magnet, before delivery of the EBRT improved the overall cancer treatment outcome. The Au part of the nanostructure is more effective for radiosensitivity, and the Fe part of the nanoparticles was effective in the physical targeting of the nanostructure. The results of blood tests showed that this treatment method had no significant toxic effects on the function of organs. Although this method of magnetic targeting has shown success in a small animal model, it must be performed in larger animals before it can be considered for future clinical applications.

## AUTHOR CONTRIBUTIONS

All authors actively participated in all phases of the study. Mahshad Mohammadkazem, Seyed M. Amini and Ali Moshiri performed the experiment and wrote the main manuscript text. Atousa Janzadeh and Ali Neshastehriz participated in the interpretation of the results and supervised the study. All authors reviewed the manuscript.

## CONFLICT OF INTEREST STATEMENT

The authors declare no conflicts of interest.

## Data Availability

All data generated or analysed during this study are included in this published article.
